# ASSOCIATION BETWEEN GRIP STRENGTH AND COGNITIVE FUNCTION AMONG COMMUNITY-DWELLING OLDER ADULTS

**DOI:** 10.1093/geroni/igz038.1797

**Published:** 2019-11-08

**Authors:** Milan Chang, Alfons Ramel, Palmi V Jonsson, Inga Thorsdottir, Olof Geirsdottir

**Affiliations:** 1 University of Iceland, School of Education, Faculty of Health Promotion, Sports and Leisure Studies, Reykjavik, Iceland; 2 University of Iceland, Reykjavik, Capital area, Iceland; 3 Landspitali University Hospital, Reykjavik, Iceland; 4 University of Iceland, Reykjavik, Iceland

## Abstract

Background: Decline in both physical function and cognition among older adults has been associated with increased risk of dementia. Physical activity (PA) is beneficial for the improvement of both physical and cognitive function. The purpose of the study was to investigate the association between baseline physical function and cognitive function after 12 weeks of resistance training among older adults. Methods: Two hundred and thirty-seven community-dwelling older adults (N=237, 73.7±5.7 years, 58.2% female) participated in a 12-week resistance exercise program (3 times/week; 3 sets, 6-8 repetitions at 75-80% of the 1-repetition maximum), designed to increase strength and muscle mass of major muscle groups. Body composition, physical activity status, grip strength, cardiovascular risk factors, 6 minutes walking distance (6MWD), and Mini-Mental State Examination (MMSE) were measured at baseline and endpoint. The linear regression model was used to examine the association. Results: Mean MMSE score was 27.5±2.1 at baseline and 28.1±2.2 after the exercise intervention. After the intervention, 57 declined, 55 remained the same, and 120 have improved in MMSE scores. We found that the MMSE score after the intervention was significantly associated with baseline grip strength (beta=.03, P<.05) among healthy older adults, after adjusting basic characteristics, cardiovascular risk factors and mobility at baseline. Conclusion: Our study found that baseline grip strength was strongly associated with cognitive function after the 12 weeks of resistance training. Muscle power, such as grip strength may play an important role in the effect of exercise intervention on cognition even among healthy independent older adults.

